# Met and its ligand HGF are associated with clinical outcome in breast cancer

**DOI:** 10.18632/oncotarget.9268

**Published:** 2016-05-10

**Authors:** Cynthia Veenstra, Gizeh Pérez-Tenorio, Anna Stelling, Elin Karlsson, Sanam Mirwani Mirwani, Bo Nordensköljd, Tommy Fornander, Olle Stål

**Affiliations:** ^1^ Department of Clinical and Experimental Medicine and Department of Oncology, Linköping University, Linköping, Sweden; ^2^ Department of Oncology-Pathology, Karolinska University Hospital and Karolinska Institute, Stockholm, Sweden

**Keywords:** radiation, copy number variation, droplet digital PCR, triple-negative breast cancer, radiotherapy

## Abstract

Few biomarkers exist to predict radiotherapy response in breast cancer. *In vitro* studies suggest a role for Met and its ligand HGF. To study this suggested role, *MET* and *HGF* gene copy numbers were determined by droplet digital PCR in tumours from 205 pre-menopausal and 184 post-menopausal patients, both cohorts randomised to receive either chemo- or radiotherapy. *MET* amplification was found in 8% of the patients in both cohorts and *HGF* amplification in 7% and 6% of the patients in the pre- and post-menopausal cohort, respectively. Met, phosphorylated Met (pMet), and HGF protein expression was determined by immunohistochemistry in the pre-menopausal cohort. Met, pMet, and HGF was expressed in 33%, 53%, and 49% of the tumours, respectively. *MET* amplification was associated with increased risk of distant recurrence for patients receiving chemotherapy. For the pre-menopausal patients, expression of cytoplasmic pMet and HGF significantly predicted benefit from radiotherapy in terms of loco-regional recurrence. Similar trends were seen for *MET* and *HGF* copy gain. In the post-menopausal cohort, no significant association of benefit from radiotherapy with neither genes nor proteins was found. The present results do not support that inhibition of Met prior to radiotherapy would be favourable for pre-menopausal breast cancer, as previously suggested.

## INTRODUCTION

Post-operative radiotherapy is an important treatment to target any remaining cancer cells in the operated area and reduce the risk of loco-regional recurrence. Although radiotherapy has a high success rate, not all breast cancer patients benefit from the treatment [1]. Accordingly, there is a need of finding new specific predictive biomarkers for radiotherapy. *In vitro* studies suggest a role for Met in radiotherapy response [2].

The Met oncoprotein is a transmembrane tyrosine kinase receptor, with major activation sites being Tyr1234/1235 in the kinase domain and Tyr1349 in the multi-docking site. The receptor is activated by extracellular binding of its ligand hepatocyte growth factor (HGF), leading to downstream signalling of pathways like the phosphoinositide 3-kinase (PI3K)/Akt pathway, stimulating tumour growth, cell survival, proliferation, migration, and invasion [3, 4]. Both Met and HGF have been shown to be involved in cancer development in several solid tumours [4–7]. The Met protein has been shown to be highly expressed in breast tumours (20-30%) and correlates with decreased survival [8–10]. Increased levels of HGF have been shown to protect cells from apoptosis [11]. Moreover, HGF can be secreted by Met positive tumour cells, creating an autocrine loop and causing a worse survival outcome [6, 12, 13].

Previous studies have shown that Met protein expression and ligand-independent activation rose after ionising radiation, and silencing of *MET* led to increased radiosensitivity [1, 2, 14]. Therefore, in the present study, it was hypothesised that the HGF/Met axis drives radioresistance in breast cancer patients, and it was aimed to determine *MET* and *HGF* gene copy number, Met/HGF expression, and Met phosphorylation in breast tumours of patients randomised to receive either chemo- or radiotherapy, in order to study correlations with clinicopathological parameters and response to radiotherapy.

## RESULTS

### *MET* and *HGF* gene copy numbers in tumours from pre- and post-menopausal patients

To establish the gene copy numbers of *MET* and *HGF* in the tumours, copy number variation assays of *MET* and *HGF* were performed with droplet digital PCR (ddPCR). *MET* amplification, defined as four or more copies, was found in 8% of the patients in both cohorts (17/205 in cohort 1, and 15/184 in cohort 2). Copy gain, defined as three or more copies, was found in 33% (66/205) and 27% (50/184) in cohort 1 and 2, respectively. *HGF* amplification was detected in 6% (11/205) and 7% (12/184) of the tumours, and copy gain in 21% (41/205) and 27% (50/184), in cohort 1 and 2, respectively. *MET* gain and *HGF* gain were significantly correlated with each other in both cohorts (p=0.01 and p<0.0001 in cohort 1 and 2, respectively).

### *MET* and *HGF* copy numbers in relation to tumour characteristics

To explore the impact of *MET* and *HGF* changed copy numbers, the genes were analysed in relation to clinicopathological parameters. For cohort 1, these are shown in Table 1 and supplementary Table S1, the correlations in cohort 2 are shown in Table 2. In both cohorts, *MET* amplification was correlated with high cell proliferation or high tumour grade. Increased *MET* copy number, either amplification or copy gain, was inversely correlated with the luminal A subtype, and thereby oestrogen receptor (ER) status, in cohort 1. In cohort 2, the same was true for *MET* amplification. In addition, *MET* amplification was more frequent in triple-negative breast cancer (TNBC) in this cohort. *HGF* copy gain was significantly correlated with a negative pAkt status in cohort 1 (supplementary Table S2). In cohort 2, an inverse correlation was found between *HGF* copies and the luminal A subtype. Tumours with increased copy number in this cohort had a significantly higher S-phase fraction (SPF) than tumours with fewer *HGF* copies.

**Table 1:** Patient characteristics and clinicopathological parameters in association with *MET* copy number and pMet expression in cohort 1

	TOTAL	*MET* AMP1			*MET* GAIN2			ALL PATIENTS	Membranous pMet			Cytoplasmic pMet		
		1-3	>3		1-2	>2			Low	High		Low	High	
	n (%)	n (%)	n (%)	P-value	n (%)	n (%)	P-value	n (%)	n (%)	n (%)	P-value	n (%)	n (%)	P-value
TOTAL	205	186 (92)	17 (8)		137 (67)	66 (33)		228	163 (75)	55 (25)		102 (47)	116 (53)	
LYMPH NODE STATUS														
0	27 (13)	20 (15)	7 (11)	0.6	25 (14)	2 (12)	0.9	29 (13)	25 (16)	2 (4)	**0.05**	15 (16)	12 (11)	0.5
1-3	112 (55)	76 (55)	34 (51)		101 (54)	9 (53)		127 (55)	90 (57)	30 (60)		54 (56)	66 (59)	
>3	66 (32)	41 (30)	25 (38)		60 (32)	6 (35)		61 (27)	42 (26)	18 (36)		27 (28)	33 (30)	
Unavailable								11 (5)						
TUMOUR SIZE (MM)														
≤20	79 (39)	54 (41)	23 (35)	0.5	73 (40)	4 (24)	0.2	87 (38)	64 (40)	21 (40)	0.9	36 (36)	49 (43)	0.3
>20	121 (59)	79 (59)	42 (65)		108 (60)	13 (76)		135 (59)	97 (60)	31 (60)		64 (64)	64 (57)	
Unavailable	5 (2)							6 (3)						
NHG														
I	47 (23)	35 (27)	12 (19)	**0.006**	44 (25)	3 (18)	**0.007**	52 (23)	41 (26)	7 (13)	0.06	20 (21)	28 (25)	**0.05**
II	104 (51)	75 (57)	28 (44)		98 (55)	5 (29)		115 (50)	81 (52)	30 (57)		46 (47)	65 (58)	
III	45 (22)	21 (16)	23 (37)		34 (20)	9 (53)		52 (23)	35 (22)	16 (30)		31 (32)	20 (18)	
Unavailable	9 (4)							9 (4)						
ER STATUS														
Negative*	54 (26)	31 (24)	22 (38)	0.06	44 (26)	9 (56)	**0.01**	62 (27)	43 (29)	14 (28)	0.9	34 (39)	23 (21)	**0.007**
Positive†	133 (65)	96 (76)	36 (62)		125 (74)	7 (44)		145 (64)	104 (71)	36 (72)		54 (61)	86 (79)	
Unavailable	18 (9)							21 (9)						
HER2 STATUS														
Negative	174 (85)	119 (88)	53 (80)	0.2	161 (87)	11(65)	**0.01**	191 (84)	142 (88)	41 (75)	**0.02**	83 (81)	100 (87)	0.3
Positive	30 (15)	17 (12)	13 (20)		24 (13)	6 (35)		35 (15)	20 (12)	14 (25)		19 (19)	15 (13)	
Unavailable	1 (0)							2 (1)						
pAkt STATUS														
Negative	101 (49)	93 (53)	7 (41)	0.4	68 (53)	32 (49)	0.7	116 (51)	95 (59)	16 (30)	**0.0002**	68 (67)	43 (38)	**0.00001**
Positive	95 (46)	84 (48)	10 (59)		61 (47)	33 (51)		105 (46)	66 (41)	38 (70)		33 (33)	71 (62)	
Unavailable	9 (5)							7 (3)						
BREAST CANCER SUBTYPE														
Luminal A	102 (50)	78 (63)	23 (41)	**0.004**	97 (59)	4 (25)	**0.02**	113 (49)	89 (62)	19 (39)	**0.005**	44 (51)	64 (60)	0.1
Luminal B1	14 (7)	6 (5)	8 (14)	**0.04**	13 (8)	1 (6)	0.8	13 (6)	4 (3)	9 (18)	**0.0002**	2 (2)	11 (10)	0.022
Luminal B2	13 (6)	10 (8)	3 (5)	0.5	11 (7)	2 (13)	0.4	14 (6)	7 (5)	7 (15)	**0.03**	6 (7)	8 (8)	0.8
HER2	12 (6)	5 (4)	7 (13)	**0.04**	8 (5)	4 (25)	**0.002**	16 (7)	10 (7)	5 (10)	0.4	10 (12)	5 (5)	0.11
TNBC	41 (20)	25 (20)	15 (27)	0.4	35 (21)	5 (31)	0.3	45 (20)	33 (23)	9 (18)	0.5	24 (30)	18 (17)	0.08
Unavailable	23 (11)							27 (12)						
ADJUVANT TREATMENT														
Chemotherapy	111 (54)	80 (58)	30 (45)	0.08	103 (55)	7 (41)	0.3	124 (54)	86 (53)	30 (55)	0.8	54 (53)	62 (53)	0.9
Radiotherapy	94 (46)	57 (42)	36 (55)		83 (45)	10 (59)		104 (46)	77 (47)	25 (45)		48 (47)	54 (47)	

Abbreviations: ER: Oestrogen receptor; NHG: Nottingham Grade; TNBC: triple-negative breast cancer. *< 0.05 fmol/μg DNA, † ≥ 0.05 fmol/μg DNA.

1Gene amplification, 2Copy Gain

**Table 2:** Patient characteristics and clinicopathological parameters in association with *MET* and *HGF* copy number in cohort 2

	TOTAL	*MET* AMP1			*MET* GAIN2			*HGF* AMP1			*HGF* GAIN2		
		1-3	>3		1-2	>2		1-3	>3		1-2	>2	
	n (%)	n (%)	n (%)	P-value	n (%)	n (%)	P-value	n (%)	n (%)	P-value	n (%)	n (%)	P-value
TOTAL	184	168 (92)	15 (8)		133 (73)	50 (27)		172 (93)	12 (7)		134 (73)	50 (27)	
LYMPH NODE STATUS														
0	20 (11)	19 (11)	1 (6)	0.5	15 (11)	5 (10)	0.4	20 (12)	0 (0)	0.4	15 (11)	5 (10)	0.3
1-3	103 (56)	96 (57)	7 (47)		78 (59)	25 (50)		96 (56)	7 (58)		79 (59)	24 (48)	
>3	61 (33)	53 (32)	7 (47)		40 (30)	20 (40)		56 (33)	5 (42)		40 (30)	21 (42)	
TUMOUR SIZE (MM)														
≤20	74 (40)	69 (41)	5 (33)	0.6	61 (46)	13 (26)	**0.01**	70 (41)	4 (33)	0.6	56 (42)	18 (36)	0.5
>20	110 (60)	99 (59)	10 (67)		72 (54)	37 (74)		102 (59)	8 (67)		78 (58)	32 (64)	
S-PHASE FRACTION														
≤10%	92 (50)	88 (58)	4 (27)	**0.02**	71 (58)	21 (47)	0.2	90 (58)	2 (17)	**0.006**	76 (62)	16 (35)	**0.01**
>10%	76 (41)	64 (42)	11 (73)		51 (42)	24 (53)		66 (42)	10 (83)		46 (38)	30 (65)	
Unavailable	16 (9)												
ER STATUS														
Negative*	53 (29)	43 (26)	9 (60)	**0.005**	33 (25)	19 (38)	0.09	47 (28)	6 (50)	0.1	31 (24)	22 (44)	0.07
Positive†	129 (70)	123 (74)	6 (40)		98 (75)	31 (62)		123 (72)	6 (50)		101 (77)	28 (56)	
Unavailable	2 (1)												
HER2 STATUS														
Negative	134 (73)	124 (75)	10 (67)	0.5	99 (75)	35 (71)	0.6	126 (75)	9 (75)	0.6	99 (75)	35 (71)	0.6
Positive	47 (26)	41 (25)	5 (33)		32 (24)	14 (29)		43 (25)	3 (25)		33 (25)	14 (28)	
Unavailable	3 (1)												
pAkt STATUS														
Negative	124 (67)	111 (67)	12 (80)	0.3	93 (70)	30 (61)	0.2	115 (68)	9 (75)	0.6	93 (70)	31 (63)	0.4
Positive	58 (32)	55 (33)	3 (20)		39 (30)	19 (39)		55 (32)	3 (25)		40 (30)	18 (37)	
Unavailable	2 (1)												
BREAST CANCER SUBTYPE														
Luminal A	64 (35)	62 (39)	2 (13)	**0.05**	51 (40)	13 (29)	0.2	63 (39)	1 (8)	**0.04**	55 (43)	9 (19)	**0.003**
Luminal B1	30 (16)	28 (18)	2 (13)	0.7	23 (18)	7 (15)	0.7	27 (16)	3 (25)	0.4	21 (16)	9 (19)	0.7
Luminal B2	28 (15)	26 (16)	2 (14)	0.8	21 (16)	7 (15)	0.8	26 (16)	2 (17)	0.9	20 (16)	8 (17)	0.9
HER2	18 (10)	14 (9)	3 (20)	0.1	10 (8)	7 (15)	0.2	16 (10)	2 (17)	0.4	12 (10)	6 (12)	0.5
TNBC	35 (19)	29 (18)	6 (40)	**0.03**	23 (18)	12 (26)	0.3	31 (19)	4 (33)	0.2	19 (15)	16 (33)	**0.007**
Unavailable	9 (5)												
ADJUVANT TREATMENT														
Tamoxifen	91 (49)	90 (54)	7 (47)	0.6	68 (51)	29 (58)	0.4	93 (54)	4 (33)	0.2	69 (51)	28 (56)	0.6
No Tamoxifen	93 (51)	78 (46)	8 (53)		65 (49)	21 (42)		79 (46)	8 (67)		65 (49)	22 (44)	
Chemotherapy	97 (53)	86 (51)	6 (40)	0.4	65 (49)	26 (52)	0.7	89 (52)	8 (67)	0.2	70 (52)	23 (46)	0.5
Radiotherapy	87 (47)	82 (49)	9 (60)		68 (51)	24 (48)		83 (48)	4 (33)		64 (48)	27 (54)	

Abbreviations: ER: Oestrogen receptor; TNBC: triple-negative breast cancer. *< 0.05 fmol/μg DNA, † ≥ 0.05 fmol/μg DNA.

1Gene amplification, 2Copy Gain

### Protein expression levels of Met and HGF in pre-menopausal patients

Protein expression levels of Met, pMet, HGF, and pAkt, a key protein in Met signalling, were studied in cohort 1 by use of immunohistochemical staining. High expression of Met in the membrane was found in 20% (45/228) of the tumours, and high cytoplasmic staining in 33% (73/228) of the cases. High pMet expression was found in 25% (55/228) and 53% (116/228) of the tumours in the membrane and the cytoplasm, respectively. High stromal HGF was found in 51% (110/228) of the tumours and high cytoplasmic staining in 49% (105/228). pAkt was highly expressed in 46% (105/228) of the tumours. Interrelationships between the proteins can be found in Supplementary Table S2. The correlations between the genes and proteins in cohort 1 can be found in supplementary Table S3.

### Met and HGF protein expression in relation to tumour characteristics

Albeit neither Met nor stromal HGF expression correlated to any of the clinicopathological parameters, membranous pMet showed a correlation with human epidermal growth factor receptor-2 (HER2) status, and cytoplasmic HGF and pMet were positively correlated with pAkt status. Both high cytoplasmic and membranous pMet were predominant in luminal B1 tumours. However, whilst membranous pMet was more often low in luminal A tumours, cytoplasmic pMet tended to be abundant in this subtype. Associations between proteins and clinicopathological parameters can be found in Table 1 and supplementary Table S1.

### Prognostic value of Met and HGF

**Figure 1:**
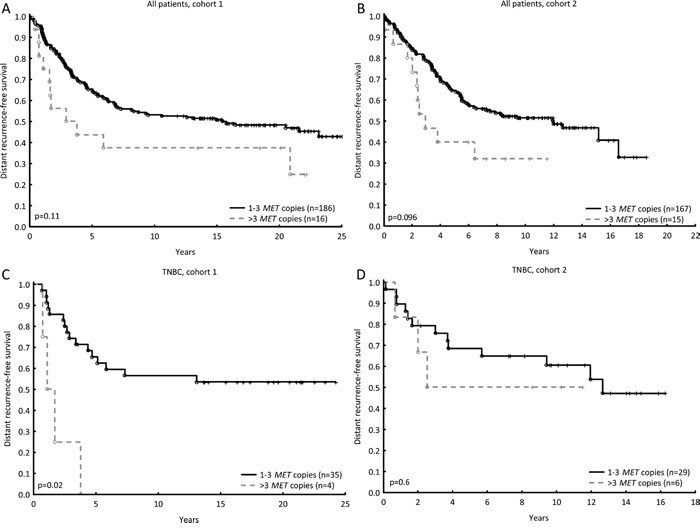
*MET* amplification (four or more copies) tended to result in a shorter DRFS in pre-menopausal patients (cohort 1, HR = 1.76; 95% CI: 0.94-3.30, p=0.08, **A**) and post-menopausal patients (cohort 2, HR = 1.9; 95% CI: 0.99-3.72, p=0.05, **B**). *MET* amplification in triple-negative breast tumours led to a shorter DRFS in pre-menopausal patients (cohort 1, HR = 6.5; 95% CI: 1.98-21.1, p=0.002, **C**), but not in post-menopausal patients (cohort 2, HR = 1.5; 95% CI: 0.43-5.5, p=0.5, **D**).

Distant recurrence-free survival (DRFS) was used as endpoint to explore the prognostic importance of the proteins and genes of interest. In both cohorts, *MET* amplification tended to result in a shorter DRFS (Figure 1A–1B). In cohort 1, but not significantly in cohort 2, *MET* amplification indicated a higher relapse rate in patients with triple-negative tumours than in TNBC patients without amplification (Figure 1C–1D). A similar result was seen in patients with *MET* gain (Hazard Ratio (HR) = 2.52; 95% confidence interval (CI): 1.04-6.1, p=0.04). This could only be seen on gene level, and not on protein level. Although *HGF* copy number was not prognostic in patients with triple-negative tumours in either of the cohorts, high stromal HGF, but not cytoplasmic HGF, was correlated with a shorter DRFS in these patients in cohort 1 (HR = 2.93; 95% CI: 1.06-8.09, p=0.04 and HR = 1.0; 95% CI: 0.4-2.4, p=0.9, respectively).

The increased rate of distant recurrence seen in relation to *MET* amplification was further explored for patients who received chemotherapy with cyclophosphamide, methotrexate, and 5-fluorouracil (CMF). In multivariable Cox regression analysis, including both cohorts adjusting for lymph node involvement, tumour size, ER and HER2 status, and stratified for cohort, *MET* amplification was associated with a significantly increased rate of distant recurrence (HR = 2.73; 95% CI: 1.36-5.5, p=0.005). Likewise, for patients with TNBC, treated with CMF, *MET* amplification showed prognostic significance with adjustments made for nodal status and tumours size (HR = 6.1; 95% CI: 1.79-20.9, p=0.004).

**Figure 2:**
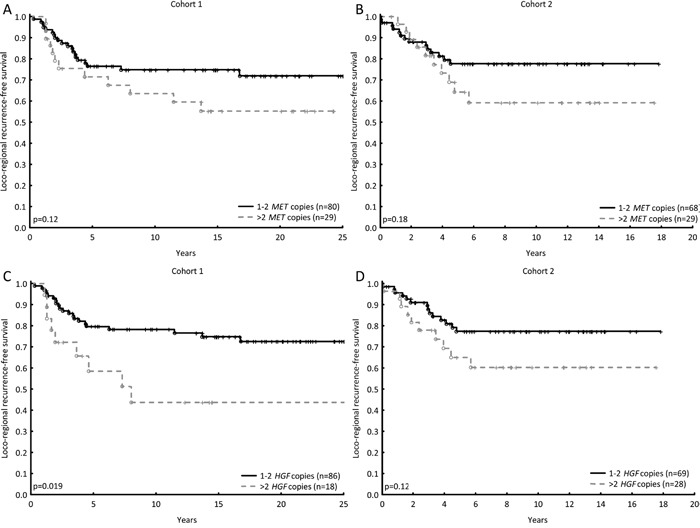
The prognostic values of *MET* and *HGF* in terms of loco-regional recurrence, for patients who did not receive post-operative radiotherapy (CMF-arm) *MET* gain (three or more copies) shows a minor trend towards higher recurrence rate in both cohorts (Cohort 1, HR = 1.8; 95% CI: 0.86-3.6, p=0.13, **A** and cohort 2, HR = 1.7; 95% CI: 0.8-3.9, p=0.18, **B**). *HGF* gain was associated with a significantly higher recurrence rate compared with no gain in cohort 1, and a similar trend in cohort 2 (HR = 2.7; 95% CI: 1.23-5.9, p=0.0014, **C** and HR = 1.9; 95% CI: 0.9-4.3, p=0.12, **D**).

As radiotherapy strongly influences loco-regional recurrence, patients who received CMF were selected to investigate the prognostic value of *MET* and *HGF* in terms of loco-regional recurrence. Patients having tumours with *HGF* gain relapsed at a higher rate than those having tumours with one or two copies of the gene in cohort 1. In cohort 2 this was merely a trend, likewise with *MET* gain in both cohorts (Figure 2). None of the proteins showed prognostic value on a loco-regional level.

### Prediction of radiotherapy benefit

**Figure 3:**
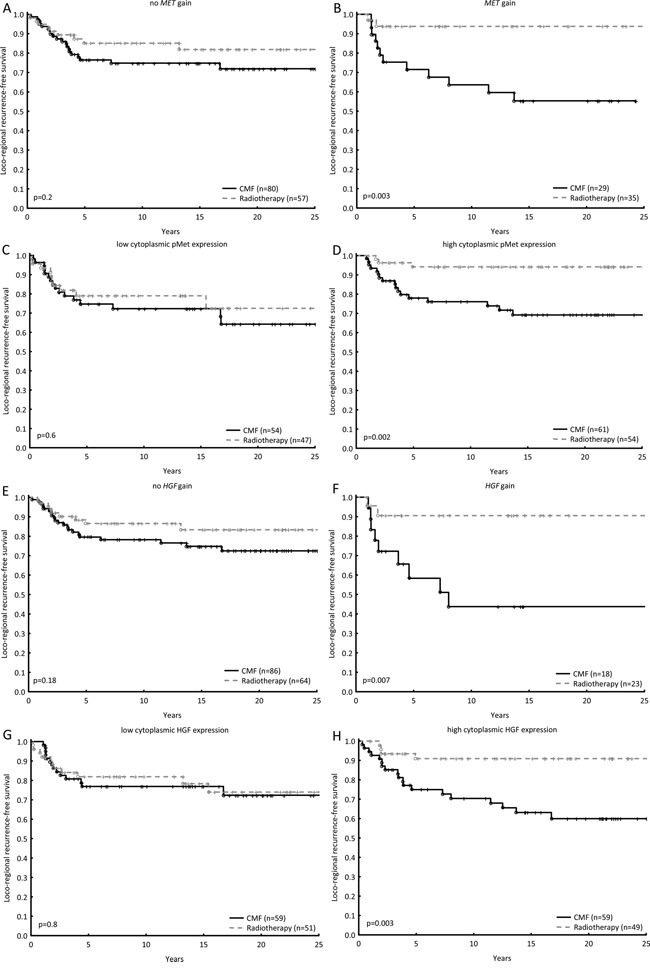
The loco-regional recurrence-free survival was estimated for patients in cohort 1 treated with radiotherapy as compared with chemotherapy in relation to no *MET* **gain and** *MET* **gain A, B.** in relation to low cytoplasmic pMet expression and high cytoplasmic pMet expression **C, D**. in relation to no *HGF* gain and *HGF* gain **E, F.** and lastly in relation to low cytoplasmic HGF expression and high cytoplasmic HGF expression **G, H.** Hazard Ratios and tests for interactions between gene/protein of interest and treatment effect can be found in Table 3.

In cohort 1, both *MET* copy gain and high cytoplasmic pMet resulted in a better response to radiotherapy than to CMF, whilst no difference in treatment benefit was detected in the case of low *MET* copy number or protein expression (Figure 3A–3D). Test for interaction showed a significant difference in response in relation to pMet (p=0.05) and a similar trend for *MET* gain (p=0.09, Table 3). Likewise, high expression of membranous pMet was correlated with a favourable response towards radiotherapy (Table 3). The same pattern was seen in cohort 1 amongst patients whose tumours harboured more than two *HGF* copies and/or high expression of cytoplasmic HGF (Figure 3E–3H, Table 3). Furthermore, similar results were obtained with pAkt status in the same cohort: high expression of pAkt led to more benefit from radiotherapy than CMF (Supplementary Figure S1). Patients in cohort 2 with *MET* gain did show more benefit from radiotherapy, though as did those with no *MET* gain. Although *HGF* gain did not demonstrate more benefit from radiotherapy, patients with no *HGF* gain did. In both cases, however, a test for interaction revealed no significant differences (Table 3).

**Table 3:** Hazard ratios for loco-regional recurrence, comparing post-operative radiotherapy and CMF, and tests for interaction between gene/protein of interest and treatment effect

	Hazard Ratio; (95% CI)	P-value for interaction	
COHORT 1			
*MET* no copy gain	0.6; 0.3-1.4, p=0.23	0.09	Figure 3A
*MET* copy gain	0.15; 0.03-0.7, **p=0.001**		Figure 3B
Low cytoplasmic pMet	0.8; 0.36-1.74, p=0.55	0.05	Figure 3C
High cytoplasmic pMet	0.18; 0.05-0.63, **p=0.007**		Figure 3D
Low membranous pMet	0.6; 0.29-1.22, p=0.15	0.2	
High membranous pMet	0.18; 0.04-0.81, **p=0.003**		
Low cytoplasmic total Met	0.42; 0.2-0.87, **p=0.02**	0.4	
High cytoplasmic total Met	0.7; 0.3-2.12, p=0.6		
Low membranous total Met	0.4; 0.22-0.87, **p=0.01**	0.3	
High membranous total Met	0.18; 0.018-1.83, p=0.15		
*HGF* no copy gain	0.6; 0.3-1.39, p=0.2	0.1	Figure 3E
*HGF* copy gain	0.16; 0.04-0.74, **p=0.019**		Figure 3F
Low cytoplasmic HGF	0.90; 0.41-1.99, p=0.8	**0.04**	Figure 3G
High cytoplasmic HGF	0.23; 0.08-0.7, **p=0.007**		Figure 3H
Low stromal HGF	0.4; 0.16-1.08, p=0.07	0.5	
High stromal HGF	0.6; 0.3-1.4, p=0.2		
COHORT 2			
*MET* no copy gain	0.35; 0.13-0.98, **p=0.05**	0.7	
*MET* copy gain	0.25; 0.05-1.14, p=0.07		
*HGF* no copy gain	0.3; 0.095-0.9, **p=0.03**	0.9	
*HGF* copy gain	0.5; 0.15-1.5, p=0.2		

## DISCUSSION

Met overexpression has in multiple studies been demonstrated to be a poor prognostic factor in breast cancer [8–10, 15–19]. Here, neither Met nor HGF expression was found to be significantly correlated with poor prognosis, even though an overexpression was observed for the proteins. It is unclear what is causing this overexpression, a common cause is known to be gene amplification. Previous studies have failed to find *MET* amplification in breast cancer [20, 21]; the present study showed *MET* amplification in only 8% of the tumours of two cohorts, and amplification did not correlate with protein expression. Although protein expression did not correlate with poor prognosis, increased *MET* copy number tended in both cohorts to be indicative of a shortened distant and loco-regional recurrence-free survival. *HGF* gain was also found to indicate loco-regional relapse at a high rate, in both cohorts. Adjuvant chemotherapy has proven to significantly improve DRFS in breast cancer. Met overexpression has recently been associated with resistance to cytotoxic drugs [22, 23]. Indeed, when including both cohorts, *MET* amplification was found to be an independent prognostic factor for patients treated with chemotherapy, and, importantly, this was also true for patients with TNBC. For this group of patients, chemotherapy is the primary systemic therapy and there is a need for finding new targets for treatment. A prognostic role for Met in TNBC was likewise found in a meta-analysis study about Met [24]. Furthermore, it was found in the pre-menopausal cohort that stromal HGF, but not tumoural HGF, was associated with shorter survival from TNBC, a difference not previously reported.

In agreement with other studies, increased *MET* copy number was shown to be inversely correlated with ER status [9, 20]. It was thereby inversely correlated with the luminal A subtype, the least aggressive of the breast cancer subtypes, indicating that Met is more prominent in more aggressive subtypes. Similarly, in cohort 2, tumours with increased *HGF* copy number tended to be ER negative. Moreover, in concordance with other studies [20, 25–27], high *MET* and *HGF* copy number in tumours from cohort 2 were associated with higher cell proliferation (SPF). The role of Met in HER2 positive breast cancer is debated. In cohort 2, no correlation was found between *MET* gain and the HER2 positive subtype, which is in agreement with the meta-analysis conducted by Yan *et al*. [24]. Cohort 1, however, showed an association between *MET* gain and the HER2 positive subtype. Another study, not included in the meta-analysis, revealed Met to be co-expressed with HER2 in breast cancer [28]. In addition, *MET* and *HGF* amplification was interrelated, which in part might be explained by the fact that both genes are located in close proximity to one another, on chromosome 7.

*In vitro*, Met and HGF have been demonstrated to negatively influence response towards ionising radiation, and it has been suggested that Met inhibition might overcome radioresistance [2, 29, 30]. Moreover, it has been demonstrated that HGF protects radiated cells from DNA fragmentation, suggestively via the PI3K/Akt pathway [31]. Whereas in a patient study on oropharyngeal cancer, Met was indeed involved in resistance to radiotherapy [32]; up to now, this has not been clinically verified in breast cancer. In the present study, it was shown that pre-menopausal patients with *MET* or *HGF* gain, or high cytoplasmic pMet or HGF expression, had more benefit from radiotherapy versus chemotherapy, as compared with those with no copy gain or low expression. This effect was not seen in post-menopausal patients, nor in relation to non-phosphorylated Met or stromal HGF in cohort 1. One confounding factor in the post-menopausal cohort might be that 50% of the patients were randomised to receive tamoxifen, known to effectively reduce loco-regional recurrence rates. It can, however, not be excluded that the HGF/Met axis differently influences pre- and post-menopausal patients and their response to radiotherapy. In cohort 2 it was previously shown that low pAkt predicted a favourable response to radiotherapy [33]. In contrast, in cohort 1 it was seen that high pAkt predicted a favourable radiotherapy response. Activation of Met may lead to activation of Akt. This, and the indication that pMet, but not total Met, led to a better radiotherapy response, suggests that an activated state of Met is of importance for the beneficial radiotherapy response.

In summary, we show that Met and HGF have a multi-factorial relationship to the biology and outcome of breast cancer, influenced by gene copy number and protein expression, activation status, stromal environment, and cellular localisation. Even though several *in vitro* studies suggest that Met inhibition might overcome radioresistance, the novel finding presented here suggests that it might not be prudent to inhibit the HGF/Met axis prior to radiotherapy, as previously suggested after *in vitro* studies. However, more research is needed to elucidate the role of phosphorylated Met and HGF in radiotherapy response.

## MATERIALS AND METHODS

### Patient material

**Figure 4:**
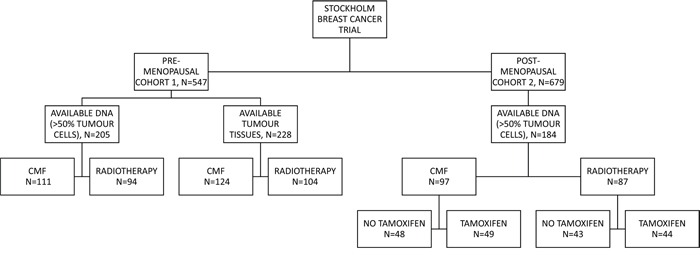
Patient distribution throughout the study The randomisation and the patient distribution is shown for both cohort 1 and 2. FFPE tissue samples were available for cohort 1, whilst fresh-frozen was available for cohort 2. CMF: cyclophosphamide, methotrexate, and 5-fluorouracil.

From 1976 to 1990, 547 pre-menopausal and 679 post-menopausal women with breast cancer in the Stockholm region were randomised to adjuvant chemotherapy or post-operative radiotherapy [34]. The post-menopausal patients were furthermore randomised to tamoxifen therapy or no adjuvant endocrine treatment, with a duration of two years. From 1983, most patients who received tamoxifen, and were disease-free for two years, were randomly assigned to stop tamoxifen treatment or to continue for three more years. Patients had either histologically verified lymph node metastasis or a tumour diameter surpassing 30 mm. Patients who received post-operative radiotherapy were given a total dose of 46 Gy, with 2 Gy per fraction for 5 days per week, targeted towards the chest wall, axilla, supraclavicular fossa, and the ipsilateral internal mammary nodes. Chemotherapy was given according to the Milan trial protocol, consisting of 12 courses of CMF (cyclophosphamide 100 mg/m^2^, orally once a day, methotrexate 40 mg/m^2^, intravenous on days 1 and 8, 5-fluorouracil 600 mg/m^2^ intravenous on days 1 and 8) [35]. Modified radical mastectomy was performed as primary surgery. Fresh frozen tissues were stored in liquid nitrogen and formalin fixed paraffin embedded (FFPE) tissues were stored at room temperature. The present study includes patients from the two cohorts originated from this trial: cohort 1 exists of pre-menopausal patients and cohort 2 of post-menopausal patients. Tumour tissue material in the form of tissue microarrays (TMA) was still available from 228 pre-menopausal patients in cohort 1 and 205 DNA samples extracted from FFPE tissue. Cohort 2 consists of 184 DNA samples extracted from frozen tumour tissue (Figure 4). All DNA samples were extracted from tumour tissue samples containing at least 50% tumour cells. DNA was stored at −70°C and during experiments at −20°C. Tumour tissue materials from cohort 1 were stored in the form of freshly cut tissue microarrays at 4°C with an extra thick layer of paraffin to reduce oxidation. Retrospective studies on archived tumour tissue, with the purpose to evaluate prognostic and treatment predicting factors, were approved by the ethics committee at Karolinska Institute in Stockholm, Sweden. The REMARK guidelines were followed in regard to the design and reportage of this study [36]. Tables 1 and 2 show the tumour and treatment characteristics of the patients included in this study. ER status in both cohorts was previously analysed by isoelectric focusing; the threshold for ER positivity was 0.05 fmol/μg DNA [37]. HER2 overexpression in cohort 1 was previously measured by immunohistochemistry (IHC), according to Herceptest Guidelines for membrane staining (Dako, Glostrup, Denmark). In cohort 2, HER2 protein expression was previously measured by flow cytometry [33]. Phospho-Akt-S473 was previously determined in cohort 2 by IHC [33]. Nottingham grade (NHG) was only available in cohort 1, S-phase fraction (SPF) was used to estimate proliferation status of the tumours in cohort 2 [38].

### DNA extraction

Genomic DNA was previously extracted from fresh frozen tumour tissues of patients in cohort 2 [39]. Genomic DNA was extracted from FFPE tumour tissues of patients in cohort 1 using the QIamp DNA FFPE Tissue Kit (Qiagen, Hilden, Germany), according to the manufacturer's protocol, with the exception of paraffin removal. The paraffin was removed with Histolab Clear (Histolab, Gothenburg, Sweden) from maximal five sections of 10 μm FFPE tissue per sample, hereafter the protocol was followed. The sample was eluted in 60 μL elution buffer and the DNA concentration was measured with QuantiFluor® ONE dsDNA Dye kit (Promega, Madison, WI, USA) on a Quantus^TM^ Fluorometer (Promega).

### ddPCR

Copy number variations were evaluated with ddPCR for *MET* and *HGF* (Bio-Rad, Hercules, CA, USA) in both cohorts, using *AP3B1* (Bio-Rad) as reference gene (Supplementary table S4). Copy number variation of *AP3B1* is reported in merely 1% of breast tumours, according to the Catalogue of Somatic Mutations in Cancer (COSMIC: http://cancer.sanger.ac.uk) and The Cancer Genome Atlas (TCGA: http://cancergenome.nih.gov), which is why it is considered a suitable reference gene. It has previously been reported that absolute gene copy numbers can be successfully determined in DNA derived from FFPE tissues, by using ddPCR [40]. The recommendations of the manufacturer were followed [41]. In short, the ddPCR reaction contained 1X ddPCR Supermix for probes (Bio-Rad), primers and probes (900 nM and 250 nM, respectively), and 10 or 5 ng DNA. Enzyme restriction with 5 units HaeIII was only performed in DNA extracted from frozen tissue (cohort 2). Droplets were generated with 20 μL ddPCR reaction mixture and 70 μL droplet generation oil (Bio-Rad). Forty μL of the generated droplets were transferred to a 96-well twin-tec PCR Plate (Eppendorf, Hamburg, Germany) and PCR was run with the following conditions: 95°C for 10 min (1 cycle), 94°C for 30s and 60°C for 60s (40 cycles), 98°C for 10 min (1 cycle) and at 4°C on hold. The ramp rate was 50%, 2°C/s and lid temperature was 105°C. Droplets were detected using Qx100 droplet reader (Bio-Rad) and data was analysed with Quantasoft v.1.3.2.0. Absolute gene copy numbers were calculated by the software as the ratio of the target molecule concentration to the reference molecule concentration, times the number of copies of the reference gene (two).

### Tissue microarray

FFPE tissues were available for the pre-menopausal cohort. Representative tissue blocks were selected as donor blocks for the TMAs. Sections were cut from each donor block and stained with Haematoxylin and Eosin. From these slides, three morphologically representative regions were chosen in all tumour samples. Three cylindrical tissue cores with a diameter of 0.8 mm were taken from these areas and mounted in recipient blocks. The TMAs were constructed using a manual arrayer (Beecher Instruments Inc., Sun Prairie, WI, USA). Five μm sections were cut from the TMA blocks and transferred to microscope slides for IHC analysis.

### Immunohistochemistry

TMA sections were stained overnight with monoclonal antibodies against Met (D1C2 XP, 1:100, Cell Signaling Technology, Beverly, MA, USA), pMet (anti-Met phospho Y1349, 1:25, Abcam, Cambridge, UK), HGF (10 μg/mL, LifeSpan Bio Sciences Inc., Seattle, WA, USA) and pAkt-S473 (D9E, 1:25, Cell Signaling). The same lot of each antibody have been used throughout this study. Sections were deparaffinised, rehydrated, and antigen retrieved using a DAKO PT module (PT Link, Dako) with DAKO PT Low (HGF, pAkt) or High (Met, pMet) pH Buffer (Envision FLEX target retrieval solution low/high pH, Dako). Serum-free protein block (Spring Bioscience, Fremont, CA, USA) was used for 60 minutes on all sections and PBS/0.1% bovine serum albumin was used for washing. After overnight incubation at 4°C with the primary antibodies, the sections were incubated at room temperature for 30 minutes with an appropriate secondary antibody (EnVision+System-HRP, Dako). Colour was developed by incubating with 3′-diaminobenzidine tetrahydrochloride (DAB/H_2_O_2_) solution, and Mayer's Haematoxylin for counterstaining (Fluka Analytical, Sigma-Aldrich). The slides were dehydrated using serial dilutions of ethanol. Images were obtained with an AxioCam ICc5 camera attached to an Axiolab A1 microscope, using Zen 2012 (blue edition) software (Zeiss, Oberkochen, Germany).

**Figure 5:**
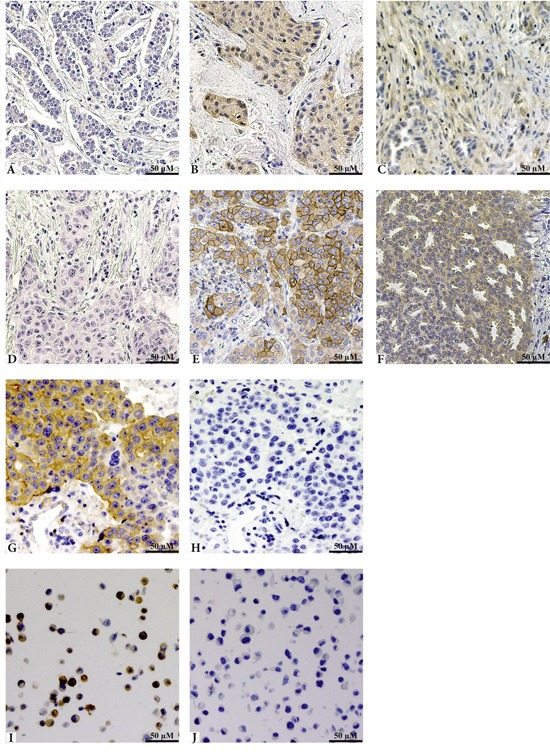
Representative images of IHC staining of HGF, pMet and the phospho-specific antibody validation Panels A through C show staining of HGF, where **A** represents negative tissue, **B** cytoplasmic staining of the tumour cells, and **C** positive staining of the stroma. Panels D to F show staining patterns of the pMet antibody and are even representative for the total Met antibody. Both antibodies showed similar staining and were graded identically. Panel **D** shows negative tissue, **E** represents membranous expression, and **F** cytoplasmic staining. **G** and **H** show staining with the pMet antibody, without and with Lambda phosphatase, respectively. Untreated MKN45 cells are shown in panel **I** and SU11274 treated MKN45 cells are shown in panel **J**, both stained with pMet.

The immunostaining was scored by two independent investigators (CV and GPT, or EK and SMM), without knowledge of clinical data. The samples were categorised into different groups. For Met and pMet, the tissues were scored following Herceptest Guidelines for membrane staining (Dako). The scoring for both the cytoplasm and membrane were divided into four groups based on intensity: negative (−), weak (+), moderate (++) and strong (+++). Met/pMet protein expression was considered high if staining was weak to strong, otherwise it was considered low. HGF was visualised in the stroma and the cytoplasm of the tumour cells. Stromal staining was either considered negative (low, -) or positive (high, +). Cytoplasmic staining was divided into negative (−), weak (+) or strong (++), based on intensity and was considered high if staining was strong. Both cell membranous and cytoplasmic staining for Met/pMet, and cytoplasmic and stromal staining for HGF were used in statistical analysis. Phospho-Akt was visualised in the cytoplasm and nucleus. Intensity was scored in the cytoplasm as negative (−), weak (+), moderate (++) or strong (+++), nuclear staining was divided in negative (−), intermediate (+), and strong (++). In this study, only cytoplasmic pAkt was used for analysis and dichotomised into low (negative to weak) and high (moderate to strong). Representative images of Met, pMet and HGF staining are shown in Figure 5A–5F. Representative images of pAkt staining can be found in a previous publication by Bostner *et al.* [42].

### Antibody validation

To verify the specificity of the Met phospho-antibody, a lambda protein phosphatase test was carried out. After pre-treatment using DAKO PT module, the slides were either treated with 4000U Lambda Protein Phosphatase (New England Biolabs, Ipswitch, MA, USA) or with washing buffer; after two hours of incubation at 30°C, the slides were treated as described above, though with an antibody dilution of 1:50. Representative images can be seen in Figure 5G&5H. To further verify the specificity of the phospho-specific antibody, a SignalSlide® (Cell Signaling), containing FFPE human gastric cancer cells MKN45, both untreated and treated with 1 μM Met inhibitor SU11274 for 2.5 hours, was stained with the pMet antibody (Figure 5I&5J). SU11274 is shown to be specific at this concentration and not able to inhibit Ron, the most closely related tyrosine kinase, which is why it is believed that inhibition is specific to Met in the used concentration [43]. Whilst cross-reactivity has not been tested, a sequence alignment showed the immunogen used for this antibody to have low homology with proteins closely related to Met: RON (0.5%), LOK (0.4%), SRC (0.7%), and RET (0.6%), indicating specificity for the pMet antibody. The anti-pAkt antibody was previously validated in our lab [42].

### Statistical analysis

Patient survival in multiple groups was computed with Kaplan-Meier and the log-rank test was carried out to estimate statistical differences. Survival time for the given end point was defined as the period of time elapsed between the diagnosis of the primary tumour and the distant recurrence (DRFS) or loco-regional recurrence (loco-regional recurrence-free survival). The Hazard Ratio was calculated using the Cox proportional hazard regression model and Pearson's chi-squared test was executed to assess the relationships between the different variables. A multivariate Cox model was used to test the interaction between benefit from post-operative radiotherapy versus chemotherapy and *MET* or *HGF* copy numbers, pAkt, pMet, Met or HGF protein expression. Multivariate Cox models were also used in the analysis of DRFS in relation to *MET* amplification and known prognostic factors, stratified for cohort.

Breast cancers were categorised into five main subtypes: Luminal A (ER+, HER2 - and Nottingham Grade (NHG) I or II or low S-phase (≤10%)); Luminal B1 (ER+, HER2- and NHG III, or high S-phase (>10%)); Luminal B2 (ER+, HER2+); HER2 positive (ER- and HER2+) and triple-negative breast cancer (TNBC) (ER- and HER2-). Progesterone receptor data was not available for the majority of the patients; hence it was not used for the categorisation of subtypes. To analyse correlations between these subtypes and either protein expression or copy number the Pearson's chi-squared test was performed for each subgroup separately.

Statistical analyses were performed with Statistica software version 12.0 (Statsoft, Tulsa, OK, USA). The criterion for statistical significance was P≤0.05 and reported in bold in the tables. Hazard-ratios were reported with 95% confidence interval.

## SUPPLEMENTARY FIGURES AND TABLES




