# Supplementary material for: Met and its ligand HGF are associated with clinical outcome in breast cancer
Source: Oncotarget. 2016 May 10;7(24):37145–59. doi: 10.18632/oncotarget.9268 (PMC5095065; doi:10.18632/oncotarget.9268)
Supplement: Supplementary file 1 [file oncotarget-07-37145-s001.pdf]

Met and its ligand HGF are associated with clinical outcome in breast cancer

SUPPLEMENTARY FIGURE AND TABLES

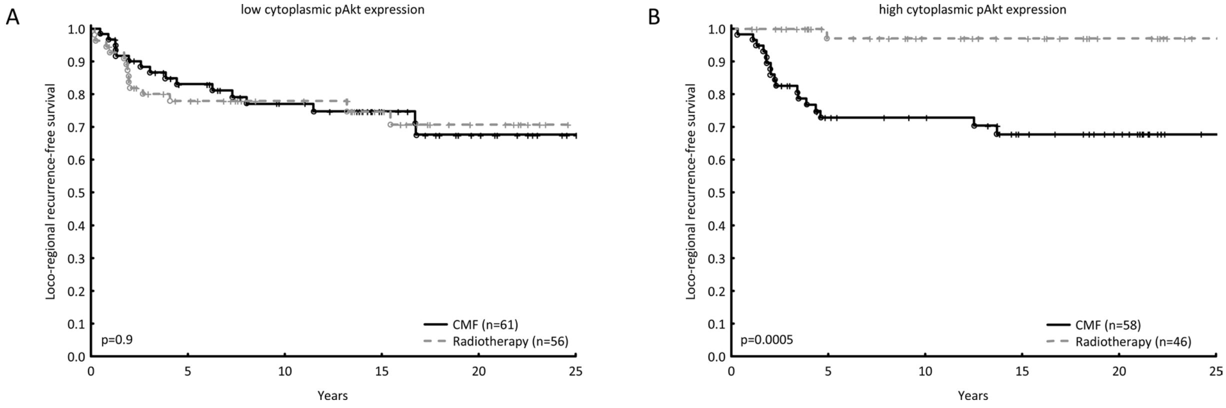

Supplementary Figure S1: The loco-regional recurrence-free survival was estimated for patients in cohort 1, treated with radiotherapy as compared with chemotherapy in relation to low cytoplasmic pAkt expression (HR = 1.1; 95% CI: 0.54-2.23, p=0.8, **A**), and high cytoplasmic pAkt expression (HR = 0.07; 95% CI: 0.009-0.5, p=0.009, **B**). Test for interaction between pAkt expression, treatment, and treatment response: p=0.0012.

SUPPLEMENTARY TABLE S1: Patient characteristics and clinicopathological parameters in association with HGF copy number, and Met and HGF expression in cohort 1

See Supplementry file 1

SUPPLEMENTARY TABLE S2: Interrelationships between Met, pMet and HGF in cohort 1

	All patients	MEMBRANOUS Met			CYTOPLASMIC Met			MEMBRANOUS pMet			CYTOPLASMIC pMet			STROMAL HGF		
		Low	High		Low	High		Low	High		Low	High		Low	High	
	n (%)	n (%)	n (%)	P-value	n (%)	n (%)	P-value	n (%)	n (%)	P-value	n (%)	n (%)	P-value	n (%)	n (%)	P-value
MEMBRANOUS Met																
Low	178 (80)															
High	45 (20)															
CYTOPLASMIC Met																
Low	73 (43)	135 (76)	15 (33)	**<0.0001**												
High	98 (57)	43 (24)	30 (67)													
MEMBRANOUS pMet																
Low	163 (75)	128 (74)	30 (73)	0.9	109 (74)	49 (74)	0.9									
High	55 (25)	44 (26)	11 (27)		38 (26)	17 (26)										
CYTOPLASMIC pMet																
Low	102 (47)	84 (49)	14 (34)	0.09	70 (48)	28 (42)	0.5	102 (64)	0 (0)	**<0.0001**						
High	116 (53)	88 (51)	27 (66)		77 (52)	38 (58)		61 (37)	55(100)							
STROMAL HGF																
Low	104 (49)	83 (49)	18 (44)	0.6	67 (47)	34 (51)	0.6	80 (52)	22 (41)	0.2	52 (53)	50 (45)	0.3			
High	110 (51)	86 (51)	23 (56)		76 (53)	33 (49)		75 (48)	32 (59)		46 (47)	61 (55)				
CYTOPLASMIC HGF																
Low	110 (51)	86 (51)	22 (52)	0.9	76 (53)	32 (47)	0.4	85 (54)	21 (39)	**0.049**	60 (61)	46 (41)	**0.003**	66 (63)	44 (40)	**0.0005**
High	104 (49)	83 (49)	20 (48)		67 (47)	36 (53)		71 (46)	33 (61)		38 (39)	66 (59)		38 (37)	66 (60)	

SUPPLEMENTARY TABLE S3: Met and HGF protein expression levels in relation to *MET* and *HGF* copy numbers

	MET AMP[1]			*MET* GAIN[2]			*HGF* AMP[1]			*HGF* GAIN[2]		
	**1-3**	**>3**		**1-2**	**>2**		**1-3**	**>3**		**1-2**	**>2**	
	**n (%)**	**n (%)**	**P-value**	**n (%)**	**n (%)**	**P-value**	**n (%)**	**n (%)**	**P-value**	**n (%)**	**n (%)**	**P-value**
MEMBRANOUS Met												
Low	140 (80)	13 (77)	0.8	104 (81)	49 (75)	0.3	135 (78)	10 (91)	0.3	114 (79)	31 (78)	0.8
High	36 (20)	4 (23)		24 (19)	16 (25)		37 (22)	1 (9)		29 (20)	9 (23)	
CYTOPLASMIC Met												
Low	118 (67)	14 (82)	0.2	88 (69)	44 (68)	0.9	114 (66)	8 (73)	0.7	93 (65)	29 (73)	0.4
High	58 (33)	3 (18)		40 (31)	21 (32)		58 (34)	3 (27)		50 (35)	11 (27)	
MEMBRANOUS pMet												
Low	127 (74)	11 (65)	0.4	96 (77)	42 (66)	0.1	127 (75)	7 (70)	0.7	105 (75)	29 (73)	0.8
High	45 (26)	6 (35)		29 (23)	22 (34)		43 (25)	3 (30)		35 (25)	11 (27)	
CYTOPLASMIC pMet												
Low	77 (45)	8 (47)	0.9	58 (46)	27 (42)	0.6	75 (44)	7 (70)	0.1	59 (42)	23 (58)	0.09
High	95 (55)	9 (53)		67 (54)	37 (58)		95 (56)	3 (30)		81 (58)	17 (43)	
STROMAL HGF												
Low	85 (50)	6 (35)	0.2	63 (51)	28 (44)	0.4	87 (52)	2 (18)	**0.03**	72 (52)	17 (43)	0.3
High	84 (50)	11 (65)		60 (49)	35 (56)		80 (48)	9 (82)		66 (48)	23 (58)	
CYTOPLASMIC HGF												
Low	88 (52)	20 (59)	0.6	67 (54)	31 (48)	0.4	90 (54)	7 (64)	0.5	71 (51)	26 (65)	0.1
High	82 (48)	7 (41)		56 (46)	33 (52)		78 (46)	4 (36)		68 (49)	14 (35)	

[1]Gene amplification, [2]Copy Gain

SUPPLEMENTARY TABLE S4: Genes and assay information as used for ddPCR

Gene	Study	Accession Number	ddPCR Assay	Fluorophore	Amplicon length (nt)
MET	Cohort 1	NM_000245.2	dHsaCP2500321	FAM	62
	Cohort 2	NM_000245.2	dHsaCP1000038	FAM	109
HGF	Cohort 1, 2	NG_016274.1	dHsaCP2500406	FAM	70
AP3B1	Cohort 1	NM_003664.3	dHsaCP2500348	HEX	60
	Cohort 2	NM_003664.3	dHsaCP1000001	HEX	85
