# Supplementary material for: Met and its ligand HGF are associated with clinical outcome in breast cancer
Source: Oncotarget. 2016 May 10;7(24):37145–59. doi: 10.18632/oncotarget.9268 (PMC5095065; doi:10.18632/oncotarget.9268)
Supplement: Supplementary file 2 [file oncotarget-07-37145-s002.docx]

# **SUPPLEMENTARY TABLE S1.** Patient characteristics and clinicopathological parameters in association with HGF copy number, and Met and HGF expression in cohort 1

|  | ***HGF* AMP1** | |  | ***HGF* GAIN2** | |  | **Membranous Met** | |  | **Cytoplasmic Met** | |  | **Stromal HGF** | |  | **Cytoplasmic HGF** | |  |
| --- | --- | --- | --- | --- | --- | --- | --- | --- | --- | --- | --- | --- | --- | --- | --- | --- | --- | --- |
|  | **1-3** | **>3** |  | **1-2** | **>2** |  | **Low** | **High** |  | **Low** | **High** |  | **Low** | **High** |  | **Low** | **High** |  |
|  | **n (%)** | **n (%)** | **P-value** | **n (%)** | **n (%)** | **P-value** | **n (%)** | **n (%)** | **P-value** | **n (%)** | **n (%)** | **P-value** | **n (%)** | **n (%)** | **P-value** | **n (%)** | **n (%)** | **P-value** |
| TOTAL | 182 (94) | 11 (6) |  | 152 (79) | 41 (21) |  | 178 (80) | 45 (20) |  | 150 (67) | 73 (33) |  | 104 (49) | 110 (51) |  | 110 (51) | 105 (49) |  |
| LYMPH NODE STATUS |  |  |  |  |  |  |  |  |  |  |  |  |  |  |  |  |  |  |
| 0 | 23 (13) | 2 (18) | 0.8 | 19 (13) | 6 (15) | 0.8 | 25 (15) | 4 (9) | 0.6 | 19 (13) | 10 (14) | 0.3 | 13 (13) | 15 (14) | 0.8 | 10 (9) | 18 (18) | 0.2 |
| 1-3 | 99 (54) | 5 (45) |  | 84 (55) | 20 (49) |  | 99 (58) | 26 (62) |  | 81 (57) | 44 (64) |  | 58 (59) | 58 (55) |  | 64 (60) | 53 (55) |  |
| >3 | 60 (33) | 4 (36) |  | 49 (32) | 15 (37) |  | 46 (27) | 12 (29) |  | 43 (30) | 15 (22) |  | 27 (28) | 32 (30) |  | 33 (31) | 26 (27) |  |
| TUMOUR SIZE (MM) |  |  |  |  |  |  |  |  |  |  |  |  |  |  |  |  |  |  |
| ≤20 | 70 (40) | 4 (36) | 0.8 | 60 (41) | 14 (35) | 0.5 | 66 (38) | 19 (42) | 0.6 | 53 (36) | 32 (44) | 0.3 | 36 (35) | 46 (43) | 0.2 | 45 (42) | 37 (36) | 0.4 |
| >20 | 107 (60) | 7 (64) |  | 88 (59) | 26 (65) |  | 107 (42) | 26 (58) |  | 93 (64) | 40 (56) |  | 67 (65) | 60 (57) |  | 63 (58) | 65 (64) |  |
| NHG |  |  |  |  |  |  |  |  |  |  |  |  |  |  |  |  |  |  |
| I | 39 (22) | 2 (20) | 0.9 | 34 (23) | 7 (18) | 0.4 | 40 (23) | 10 (23) | 0.9 | 29 (20) | 21 (30) | 0.5 | 26 (25) | 21 (20) | 0.1 | 28 (26) | 20 (20) | 0.1 |
| II | 96 (55) | 5 (50) |  | 82 (56) | 19 (50) |  | 89 (52) | 23 (54) |  | 80 (56) | 32 (45) |  | 56 (55) | 53 (51) |  | 58 (54) | 51 (51) |  |
| III | 40 (23) | 3 (30) |  | 31 (21) | 12 (32) |  | 42 (25) | 10 (23) |  | 34 (24) | 18 (25) |  | 20 (20) | 30 (29) |  | 21 (20) | 29 (29) |  |
| ER STATUS |  |  |  |  |  |  |  |  |  |  |  |  |  |  |  |  |  |  |
| Negative* | 47 (28) | 3 (30) | 0.9 | 39 (28) | 11 (31) | 0.8 | 43 (27) | 17 (40) | 0.1 | 35 (26) | 25 (37) | 0.09 | 25 (25) | 31 (33) | 0.2 | 29 (29) | 28 (30) | 0.9 |
| Positive† | 119 (72) | 7 (70) |  | 101 (72) | 25 (69) |  | 117 (73) | 26 (60) |  | 101 (74) | 42 (63) |  | 75 (75) | 63 (67) |  | 72 (71) | 66 (70) |  |
| HER2 STATUS |  |  |  |  |  |  |  |  |  |  |  |  |  |  |  |  |  |  |
| Negative | 153 (85) | 11 (100) | 0.2 | 129 (85) | 35 (85) | 0.9 | 147 (83) | 40 (91) | 0.2 | 129 (86) | 58 (82) | 0.4 | 89 (86) | 91 (83) | 0.7 | 94 (85) | 87 (84) | 0.7 |
| Positive | 28 (15) | 0 (0) |  | 22 (15) | 6 (15) |  | 30 (17) | 4 (9) |  | 21 (14) | 13 (18) |  | 15 (14) | 18 (17) |  | 16 (15) | 17 (16) |  |
|  |  |  |  |  |  |  |  |  |  |  |  |  |  |  |  |  |  |  |
| pAkt STATUS |  |  |  |  |  |  |  |  |  |  |  |  |  |  |  |  |  |  |
| Negative | 89 (51) | 6 (55) | 0.8 | 68 (47) | 27 (69) | **0.01** | 93 (53) | 19 (45) | 0.4 | 77 (53) | 35 (49) | 0.6 | 53 (51) | 58 (53) | 0.7 | 70 (64) | 41 (39) | **0.002** |
| Positive | 85 (49) | 5 (45) |  | 78 (53) | 12 (31) |  | 82 (47) | 23 (55) |  | 69 (47) | 36 (51) |  | 51 (49) | 51 (47) |  | 39 (36) | 64 (61) |  |
| BREAST CANCER SUBTYPE |  |  |  |  |  |  |  |  |  |  |  |  |  |  |  |  |  |  |
| Luminal A | 90 (56) | 5 (56) | 0.9 | 76 (56) | 19 (54) | 0.8 | 89 (57) | 22 (54) | 0.8 | 81 (61) | 30 (47) | 0.06 | 61 (62) | 46 (51) | 0.06 | 60 (61) | 47 (51) | 0.3 |
| Luminal B1 | 13 (8) | 1 (11) | 0.8 | 10 (7) | 4 (11) | 0.5 | 11 (7) | 2 (5) | 0.7 | 9 (7) | 4 (6) | 0.8 | 6 (6) | 6 (7) | 0.96 | 5 (5) | 7 (8) | 0.5 |
| Luminal B2 | 13 (8) | 0 (0) | 0.4 | 12 (9) | 1 (3) | 0.2 | 13 (8) | 1 (2) | 0.2 | 8 (6) | 6 (9) | 0.4 | 6 (6) | 8 (8) | 0.6 | 5 (5) | 9 (10) | 0.3 |
| HER2 | 10 (6) | 0 (0) | 0.4 | 7 (5) | 3 (9) | 0.5 | 12 (8) | 3 (7) | 0.9 | 10 (7) | 5 (8) | 0.9 | 8 (8) | 7 (8) | 0.7 | 7 (7) | 8 (9) | 0.8 |
| TNBC | 36 (22) | 3 (33) | 0.5 | 31 (23) | 8 (23) | 0.9 | 31 (20) | 13 (32) | 0.09 | 25 (19) | 19 (30) | 0.1 | 17 (17) | 24 (26) | 0.2 | 22 (22) | 20 (22) | 0.96 |
| ADJUVANT TREATMENT |  |  |  |  |  |  |  |  |  |  |  |  |  |  |  |  |  |  |
| Chemotherapy | 101 (55) | 4 (36) | 0.2 | 87 (53) | 18 (44) | 0.1 | 95 (54) | 26 (58) | 0.6 | 77 (51) | 44 (60) | 0.2 | 54 (52) | 60 (55) | 0.7 | 59 (54) | 55 (52) | 0.9 |
| Radiotherapy | 81 (45) | 7 (64) |  | 65 (43) | 23 (56) |  | 83 (46) | 19 (42) |  | 73 (49) | 29 (40) |  | 50 (48) | 50 (45) |  | 51 (46) | 50 (48) |  |
| Abbreviations: ER: Oestrogen receptor; NHG: Nottingham Grade; TNBC: triple-negative breast cancer. *< 0.05 fmol/µg DNA, † ≥ 0.05 fmol/µg DNA.  ^1^Gene amplification, ^2^Copy Gain | | | | | | | | | | | | | | | | | | |
|  | | | | | | | | | | | | | | | | | |  |
